# Le nœud ileosigmoidien: à propos de 2 cas

**Published:** 2012-03-09

**Authors:** Fatimazahra Bensardi, Khalid Elhattabi, Abdelaziz Fadil, Nadia Benissa, Rachid Lefriyekh, Driss Khaiz, Saad Berrada, Ouariti Najib Zerouali

**Affiliations:** 1 Service des urgences chirurgicales viscérales – CHU Ibn Rochd, Casablanca, Maroc

**Keywords:** Nœud iléosigmoïdien, scanner abdominal, occlusion intestinal, nécrose

## Abstract

Le nœud iléosigmoïdien ou l’ileosigmoid knot (ISK), est une entité clinique exceptionnelle, c’est une urgence chirurgicale caractérisée par une strangulation du grêle en formant un nœud autour de la base du colon sigmoïde avec risque de nécrose rapide du grêle et du colon. Nous rapportons deux cas dans le service des urgences chirurgicales viscérales du CHU Ibn Rochd de Casablanca ; chez lesquels le diagnostic d’ISK n’a été posé qu’en peropératoire. A travers ces 2 cas et une revue de la littérature nous allons définir les aspects diagnostiques, thérapeutiques et pronostiques de cette entité clinique rare.

## Introduction

Le nœud iléosigmoïdien est une cause rare de strangulation de l’intestin grêle, ce dernier tourne autour de la base du colon sigmoïde et forme un nœud occasionnant ainsi une obstruction du sigmoïde. Cette entité clinique est exceptionnelle dans les pays de l’ouest, mais elle est commune dans certaines nations africaines, asiatiques du moyen-est [1,2]. Le nœud iléosigmoïdien est considéré comme une vraie urgence chirurgicale qui évolue rapidement vers la nécrose intestinale. Ainsi La connaissance du mécanisme de cette pathologie est essentielle, afin de pouvoir poser un diagnostic précoce et permettre une prise en charge chirurgicale rapide.

## Patients et observations

### Cas 1

**Figure 1:**
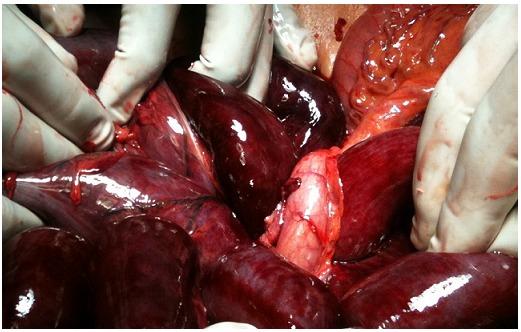
Vues per-opératoires du nœud iléal autour de la base du sigmoïde

Un homme de 55 ans, sans antécédent chirurgical, admis aux urgences, pour un syndrome occlusif fait de vomissement et arrêt des matières et des gaz, évoluant depuis 5j. A l’admission malade en état de choc hémodynamique, hypotherme à 36°c, TA=7/4 mmHg, abdomen sensible à la palpation, orifices herniaires libres, toucher rectal ampoule vide. Acheminé directement au bloc après correction hémodynamique, et sous couverture antibiotique, le malade fut opéré, l’exploration chirurgicale avait retrouvé un liquide de souffrance intestinale de grande abondance qui a été évacué; avec un volvulus du grêle autour du sigmoïde sous forme de nœud, avec nécrose iléale sur 2m à 20cm de la jonction iléocœcale et nécrose de la boucle sigmoïdienne (Figure 1). Le malade a bénéficié d’une résection en monobloc du grêle du sigmoïde nécrosés, avec une double stomie iléale droite et colostomie iliaque gauche, car toute anastomose était risquée en raison de l’hypovolémie profonde et pour minimiser le temps opératoire. Séjour en réanimation en postopératoire pendant 4j, avec transfusion de 4 culots globulaires, 4 culots plaquettaires et 6 plasma frais congelés, Les suites opératoires étaient simples, le patient est sorti à J6 postopératoire. Un mois après le malade a été opéré pour rétablissement de la continuité colique et grêlique.

### Cas 2

**Figure 2:**
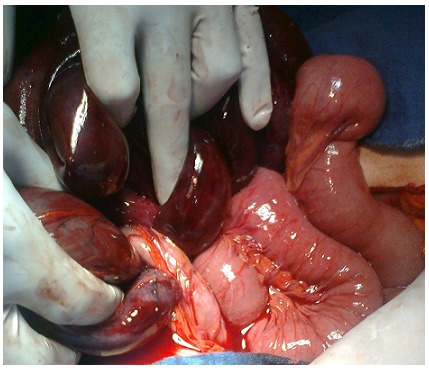
Autre vues per-opératoires du nœud iléal autour de la base du sigmoïde

**Figure 3:**
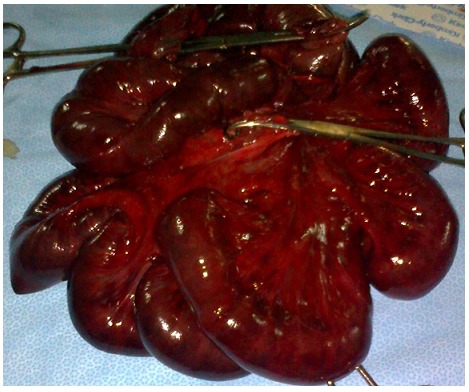
Pièce chirurgicale de la résection en monobloc du grêle et du colon nécrosés

Une femme de 29 ans, primipare, admise à j2 du post-partum (voie basse) pour syndrome occlusif. A l’examen clinique la patiente était anxieuse, asthénique avec polypnée et tachycardie, température à 37°c (sous antibiotiques), TA= 10/6 mmHg. L’abdomen tympanique, sensible à la palpation. Touchers pelviens sans particularité. L’ASP a montré des niveaux hydro-aériques de type grêlique et colique avec grisaille diffuse. L’échographie abdominopelvienne a révélé un épanchement de grande abondance échogène. La patiente fut opérée, l’exploration a retrouvé une péritonite stercorale très évoluée suite à une perforation et nécrose grêlique étendue sur 80cm, à 15cm du carrefour iléo-caecal, secondaire à un nœud iléosigmoïdien, le colon était aussi nécrosé (Figure 2). Le geste chirurgical a consisté en une toilette péritonéale abondante, résection en monobloc du tube digestif nécrosé (Figure 3), avec iléostomie droite vue les conditions septiques; et colostomie gauche type Hartmann car extension basse de la nécrose sur le rectum. Les suites postopératoires en réanimation étaient simples. La malade est sortie à j6 postopératoire, puis réadmise un mois après pour rétablissement de la continuité colique et grêlique.

## Discussion

Le nœud iléosigmoïdien ou double volvulus iléosigmoïdien, est une urgence chirurgicale rare, qui touche surtout l’homme à la quatrième décade [3,4]. Il survient dans la majorité des cas quand une boucle d’anse grêlique descend dans la gouttière paracolique gauche et tourne autour de la base du colon sigmoïde dans le sens horaire ou antihoraire et forme un nœud. Cependant dans une minorité des cas le sigmoïde peut être le composant actif dans la survenue de l’obstruction en augmentant le péristaltisme avec une torsion des deux portions d’intestins et une double strangulation. En fonction de la force de serrage du nœud et de l’engagement du mésentère, cette compression peut causer rapidement la nécrose ischémique de l’iléon et du colon (74-80%) [1–5]. Cette nécrose peut s’étendre jusqu’à l’iléon terminal et même parfois atteindre le cœcum ou le colon ascendant. De ce fait il est primordial de poser le diagnostic précocement. Alver et al [5] a classé le nœud iléosigmoidien en 4 types: type I le plus fréquent survient quand l’iléon (composant actif) tourne autour du sigmoïde; type Ia quand la torsion se fait dans le sens horaire, type Ib sens antihoraire. Type II le colon sigmoïde (composant actif) pivote autour de l’iléon. Type III quand l’iléo-cœcum tourne autour du sigmoïde. Et indéterminée quand c’est difficile de préciser le composant actif et passif [5,6].

Le diagnostic préopératoire est difficile, possible dans moins de 20% des cas en raison de sa rareté et d’une atypie clinico-radiologique [1,2,7]. Initialement le patient se présente pour des douleurs abdominales aigues centrales, qui peuvent parfois le réveiller du sommeil, le patient peut préciser l’heure exacte de survenue, puis la douleur devient constante et généralisée associée à des vomissements. Dans 56% des cas, le patient se présente avec un état de choc hypovolémique [2,3,7]. L’examen abdominal révèle une distension modérée de l’abdomen, avec une sensibilité ou une défense abdominale à la palpation, avec silence intestinale quand la nécrose intestinale est déjà installée. [7,8]. L’ASP peut montrer une distension disproportionnée comportant des niveaux hydro-aériques larges dans le colon sigmoïde occupant le côté droit de l’abdomen, avec multiples niveaux hydro-aériques de l’intestin grêle au niveau du côté gauche de l’abdomen. Le scanner abdominal peut aider au diagnostic en montrant le signe de tourbillon avec une déviation médiane du cœcum, du colon descendant et des vaisseaux mésentériques supérieurs et inférieurs qui vont converger vers ce tourbillon. Une distension intestinale ainsi que des signes d’ischémie intestinale à un stade tardif [6,8,9]. Devant ces données radiologiques d’occlusion du colon associées à la triade clinique de l’occlusion grêlique, le diagnostic de nœud iléosigmoïdien est plausible dans 71% des cas. Il est essentiel de le différencier du volvulus du sigmoïde car la réduction endoscopique est contre-indiquée.

Le deuxième cas de nœud iléosigmoïdien que nous rapportons, est survenu en postpartum chez notre patiente ; dans notre connaissance peu de cas ont été publié dans la littérature. La particularité, c’est que la grossesse rend difficile le diagnostic d’occlusion intestinale, surtout quand la parturiente rentre déjà en période de travail ; c’est pourquoi le diagnostic est généralement posé tardivement en postpartum ; et en conséquence la nécrose intestinale est déjà installée, avec une mortalité maternelle (20%) et fœtale (24-31%) non négligeable [10]. En conclusion, il ne faut pas hésiter à suspecter le diagnostic d’occlusion intestinale en période de grossesse quand il y’a des signes d’appels. Après stabilisation hémodynamique, la laparotomie en urgence ne doit pas être retardée elle confirme le diagnostic. La procédure chirurgicale idéale en cas de nœud iléosigmoïdien est un sujet à controverse. Quand l’intestin est viable, certains auteurs optent pour la tentation de levée simple du nœud, d’autres préfèrent la résection du colon sigmoïde pour prévenir les récidives. En cas de nécrose intestinale une résection du grêle, du colon et du nœud en monobloc est recommandée. Une anastomose grêle-grêlique termino-terminale ou latéro-cœcale est la règle, associé à une anastomose colo-colique termino-terminale si les conditions locales et générales le permettent, sinon une colostomie iliaque gauche est de mise [4,5,7].

## Conclusion

Seul un diagnostic préopératoire précoce avec une prise en charge chirurgicale rapide, peuvent améliorer le pronostic de cette pathologie, en diminuant la morbi-mortalité due au retard diagnostique.
